# Factors Contributing to Delays to Accessing Appendectomy in Low- and Middle-Income Countries: A Scoping Review

**DOI:** 10.1007/s00268-023-07183-2

**Published:** 2023-09-25

**Authors:** Johnelize Louw, M. McCaul, R. English, P. S. Nyasulu, J. Davies, C. Fourie, J. Jassat, K. M. Chu

**Affiliations:** 1 Centre for Global Surgery, Department of Global Health, Faculty of Medicine and Health Sciences, Stellenbosch University, Cape Town, South Africa; 2 Division of Health Systems and Public Health, Department of Global Health, Faculty of Medicine and Health Sciences, Stellenbosch University, Cape Town, South Africa; 3 Division of Epidemiology and Biostatistics, Department of Global Health, Faculty of Medicine and Health Sciences, Stellenbosch University, Cape Town, South Africa; 4 Faculty of Medicine and Health Sciences, Stellenbosch University, Cape Town, South Africa; 5 Division of Epidemiology and Biostatistics, School of Public Health, Faculty of Health Sciences, University of the Witwatersrand, Johannesburg, South Africa; 6 Department of Surgery, University of Botswana, Plot 4775 Notwane Rd, Gaborone, Botswana

## Abstract

**Background:**

Appendicitis is one of the most common emergency surgical conditions worldwide. Delays in accessing appendectomy can lead to complications. Evidence on these delays in low- and middle-income countries (LMICs) is lacking. The aim of this review was to identify and synthesise the available evidence on delays to accessing appendectomy in LMICs.

**Methods:**

This scoping review followed the Preferred Reporting Items for Systematic Reviews and Meta-analysis Extension for Scoping Reviews framework. The delays and their interconnectivity in LMICs were synthesised and interpreted using the Three Delays framework. We reviewed Africa Wide EBSCOhost, PubMed–Medline, Scopus, Web of Science, African Journals Online (AJOL), and Bioline databases.

**Results:**

Our search identified 21 893 studies, of which 78 were included in the final analysis. All of the studies were quantitative. Fifty per cent of the studies included all three types of delays. Delays in seeking care were influenced by a lack of awareness of appendicitis symptoms, and the use of self and alternative medication, which could be linked to delays in receiving care, and the barrier refusal of medical treatment due to fear. Financial concerns were a barrier observed throughout the care pathway.

**Conclusion:**

This review highlighted the need for additional studies on delays to accessing appendectomy in additional LMICs. Our review demonstrates that in LMICs, persons seeking appendectomy present late to health-care facilities due to several patient-related factors. After reaching a health-care facility, accessing appendectomy can further be delayed owing to a lack of adequate hospital resources.

**Supplementary Information:**

The online version contains supplementary material available at 10.1007/s00268-023-07183-2.

## Background

A third of the global burden of disease can be treated through surgical care [1]. Yet access is inequitable in and between low- and middle-income countries (LMICs), where 90% of people lack access to timely surgical care [1, 2]. Appendicitis, the most common surgical emergency worldwide, is a time-sensitive disease and can lead to high rates of morbidity and mortality if left untreated [1, 3, 4]. Although males and females of any age and all races can be affected, the disease is more prevalent in children and young adults [5, 6]. Africa and South-East Asia have the highest unmet surgical need for appendectomy [1]. Appendectomies are more commonly used to manage appendicitis in LMICs rather than antibiotics as patients tend to present late [7]. There are several reasons for this delay in presentation.

Delays to appendectomy care have been described in the context of different frameworks [8]. Our review will use the Three Delays framework [8], which includes seeking (Delay 1), reaching (Delay 2), and receiving care (Delay 3) [8–10]. The Lancet Commission on Global Surgery recommended this framework to quantify delays in emergency surgical care [9]. This comprehensive framework allows all factors associated with delays to care, including social, economic, health system, and environmental factors, to be examined and was therefore chosen.

There is a need to synthesise the available evidence at all points in the appendectomy care pathway to enable the design of targeted interventions. To this end, the aim of this study was to identify and synthesise the available evidence on delays to accessing appendectomy in LMICs, in the context of the Three Delays framework.

## Methodology

### Study design

This scoping review used the Preferred Reporting Items for Systematic Reviews and Meta-analysis Extension for Scoping Reviews (PRISMA-ScR) [11] and the Arksey & O’Malley framework [12, 13]. The search strategy was designed using the PEO (Population/Exposure/Outcome) framework. The elements were defined as: P (people who had appendectomy in LMICs), E (factors associated with a delay to appendectomy in LMICs), and O (delays to appendectomy experienced in LMICs).

### Eligibility criteria

The search period was January 1990–January 2022. Studies published before the year 1990 were excluded due to their temporal irrelevance. To be eligible for inclusion, studies had to either note that there was a delay to, and, or list factors that led to a delay in accessing appendectomy within a LMIC. Included studies could be cross-sectional, case–control, case series, cohort, interventional, qualitative, or mixed methods.

Studies were excluded if it focussed on the diagnosis, pathophysiology or aetiology of appendicitis, post-operative or non-operative management of appendicitis, duplicate studies, or high-income countries (HICs). We excluded case reports, editorials, commentaries, books, reviews, and study protocols. Studies not published in English, and that could not be translated using Google Translate, as well as those where the full-text versions were unavailable, were excluded.

### Searching electronic databases

Databases searched were Africa-Wide EBSCOhost, PubMed–Medline, Scopus, Web of Science, African Journals Online (AJOL), and Bioline. The search strategy comprised of the World Bank list of LMICs combined with the Medical Search Headings (MeSH) for appendicitis and appendectomy, and the truncated elements appendi* and appendec*. Synonyms and different spellings of appendectomy were included in the search string. Keywords for similar concepts were combined using the Boolean operator “OR”, and different concepts were combined with “AND”. An example of our search strategy used for one of the databases can be seen in Supplementary Table 1.

### Searching other sources

Reference lists of relevant studies, reviews, and grey literature were hand searched for additional studies that fit the PEO framework.

### Screening and study selection

Studies were imported into Covidence review software (Veritas Health Innovation, Melbourne, Australia), which was used to perform our review. All studies were reviewed in duplicate, and all reviewers discussed results at weekly conflict resolution meetings. Discordant results were discussed by the primary review team (JL, JD, JJ, CF), and the eligibility criteria were re-reviewed for individual conflicts. Senior authors (MM, KC) resolved any final conflicts.

### Data extraction, chartering, and analysis

The extraction of the included studies was done in duplicate using a standard template [12] which was edited to ensure alignment with the study objectives. In the end, the primary author reviewed and collated the captured information.

Data were captured into Microsoft Excel. Variables collected included author name(s), study title, publication year, country, aim, design, timeframe, and the type of delay and any associated factors. Data were analysed and summarised using STATA Statistical software, version 15 (Stata Corp LP, College Station, Texas, USA). No inferential or hypothesis testing was conducted. Delays to appendectomy and the associated factors were categorised using the Three Delays framework. Our review captured factors that affected health-seeking behaviour (Delay 1), factors that influenced reaching a facility (Delay 2), and factors that influenced receiving care at a health-care facility (Delay 3). There was no limit to the number of delays and associated factors that could be extracted from each study. Factors linked to each delay were mapped. Additionally, factors that linked to more than one delay were considered “interconnected” and were also mapped.

## Results

### Search results

**Fig. 1:**
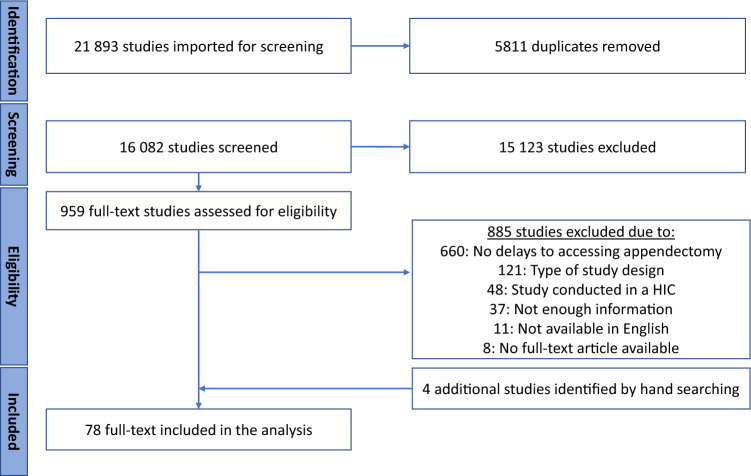
The PRISMA-ScR flowchart

Our search yielded 21 893 studies of which 5 811 were removed as duplicates, leaving 16 082 for title and abstract screening. Based on the eligibility criteria, 885 full-text studies were excluded. The most common reasons for full-text exclusion were a lack of description of appendectomy delay factors (n = 660) and the type of study design (n = 121) which comprised of 64 reviews, 26 editorials, 13 case reports, 11 conference abstracts for which no full-text article was available, 3 study protocols, 2 books, 1 dissertation, and 1 retracted article (Fig. 1). In the end, 78 studies were included in our review. Detailed descriptions of these studies can be found in Supplementary Table 2.

### Study characteristics

**Fig. 2:**
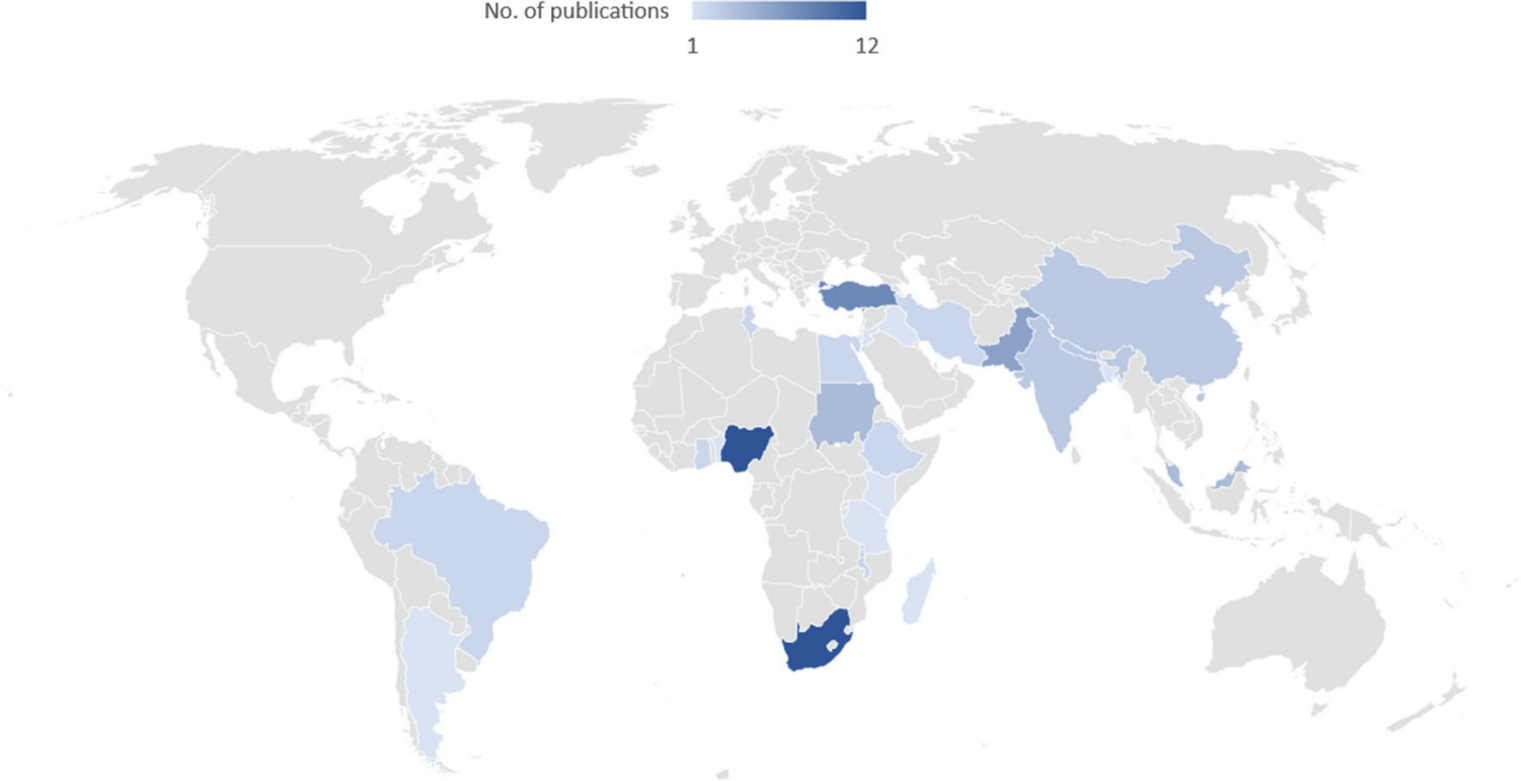
The study distribution according to WHO region and country

The three LMICs with the highest number of publications were South Africa (n = 12), Nigeria (n = 12), and Turkey (n = 8) (Fig. 2). All the studies were quantitative. The most common study designs were cross-sectional 38 (48.7%) [14–52] and cohort 32 (41.0%) [26, 52–83] (Table 1). Study periods ranged from two months to twenty-five years. There were 27 097 patients across the seventy-eight studies with a median of 166 (IQR: 10–3717) [14–28, 30, 32–42, 44–61, 63, 64, 66–82, 84–92].

**Table 1:** Types of studies

Study design	Prospective (n, %)	Retrospective (n, %)
Interventional–non-randomised control trial	1 (3.7)	0 (0)
Case series	2 (7.4)	4 (7.8)
Cross-sectional	15 (55.6)	23 (45.1)
Case-control	0 (0.0)	1 (2.0)
Cohort	9 (33.3)	23 (45.1)
Total	27 (100)	51 (100)

### Study outcomes measured

Fifty-nine of the studies [15, 17, 18, 20, 22, 24, 25, 27, 28, 30–37, 39–41, 44, 45, 47, 49–56, 58–60, 63–72, 74–76, 78–81, 83, 86, 88–90] reported perforation rates in 3603 (25.9%) patients, fifteen studies [14, 16, 19, 26, 29, 42, 46, 48, 57, 61, 62, 84, 85, 87, 92] reported the complication rate for 2071 (41.2%) patients, and four of the studies [23, 38, 43, 77] did not report either. In addition, fifty-five studies reported mortality rates. Of these studies, 141 (0.8%) appendicitis patients died [14–16, 18, 21, 22, 24–27, 29–34, 36, 37, 39–41, 44–46, 48, 51, 52, 59–67, 69–72, 74, 77, 78, 82, 85–89].

### Delays to accessing appendectomy

**Fig. 3:**
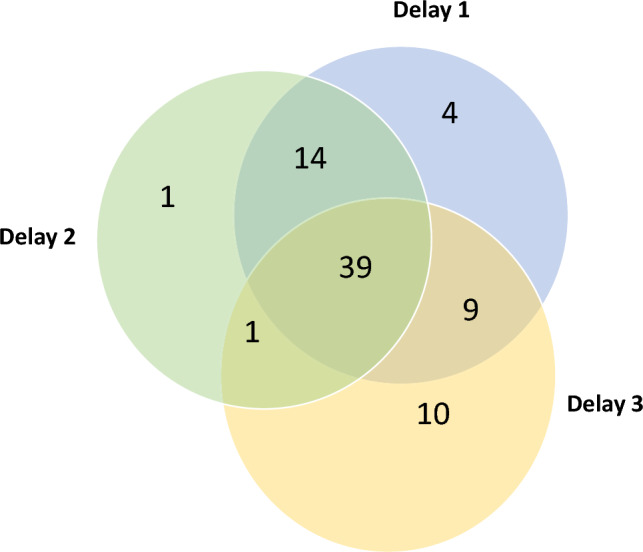
The overall number of studies (n) reporting Delay 1 (seeking care), Delay 2 (reaching care), and/or Delay 3 (receiving care)

Thirty-nine (50%) studies reported on all three delays, twenty-four reported at least two delays, and fifteen reported on only one delay (Fig. 3).

### Factors associated with delay 1 (seeking care)

#### Lack of knowledge of appendicitis symptoms and alternative forms of health-care

Thirty-five studies reported that a lack of knowledge of appendicitis symptoms led to a delay in seeking appendectomy [14, 16, 17, 19–23, 30–32, 39–42, 44, 45, 48, 49, 52, 53, 55, 56, 59, 65, 70, 71, 73–75, 77–79, 83, 89] (Table 2). The inability of vulnerable populations to communicate their symptoms to their parents or caregivers can lead to a delay in seeking care [22, 49]. Elderly appendicitis patients may have initially related their acute symptoms to existing co-morbidities, while pregnant women related their symptoms to pregnancy [48, 86]. Moreover, twenty-one studies reported that appendicitis patients first considered alternative forms of health-care such as self-medication [14, 17, 19, 21, 22, 25, 42, 45, 56, 71, 74, 75, 77, 83], visiting a traditional healer [23, 32, 38, 39, 75, 78], or allied health professionals [16], prior to seeking care in the formal health sector (Table 2).

**Table 2:** Factors leading to delays to appendectomy

Factor leading to delays	Number of studies (n)
Delays in seeking health-care	
Lack of knowledge of appendicitis-like symptoms and alternative forms of health-care	35
Financial concerns and personal factors	20
Gender and cultural disparities	7
Delays in reaching care	
Distance to health-care facilities	15
Lack of adequate transport and infrastructure including financial challenges	14
Delays in receiving care	
Delay in diagnosing and managing appendicitis	48
Lack of infrastructure	18
Lack or shortage of hospital resources	16
Cultural and financial factors that affect receiving care	6

#### Financial concerns and personal factors

Fourteen studies reported that financial concerns influenced the decision to seek care (Table 2) [19, 21, 30, 35, 39, 50, 53, 55, 59, 71, 73, 77, 87, 88]. In two studies, farmers and daily wage earners in rural communities only sought care for appendicitis when they were severely ill [19, 87]. Nine studies [19, 21, 42, 55, 59, 65, 70, 74, 87] reported personal factors leading to a delay in seeking care such as the fear of hospitalisation or undergoing surgery [21, 32, 35, 65, 74]. During the COVID-19 pandemic, seeking care was delayed due to the fear of contracting the virus or compliance with government regulations (Table 2) [29, 31, 81].

#### Gender and cultural disparities

Six studies noted that gender and cultural disparities influenced the decision to seek care for appendectomy (Table 2) [17, 20, 36, 50, 54, 77]. Two studies reported that females seek care for abdominal complaints sooner than men [17, 54]. However, often women have to consult their husbands prior to seeking care and can then only do so with their husband’s permission [20, 77]. In eastern Turkey, one of the studies reported that boys were a higher priority than girls, and that girls living in these rural regions hid their appendicitis symptoms from their parents [20].

### Factors associated with delay 2 (reaching care)

#### Distance to health-care facilities

Eight studies reported that distance can affect individuals’ ability to reach a health-care facility (Table 2) [23, 26, 37, 53, 59, 65, 77, 82]. Overall, rural dwellers presented later to health-care facilities than urban dwellers, thus increasing their risk of complications [37, 59, 61, 65].

#### Lack of adequate transport and transport infrastructure

Poor roads, difficult terrain, underdeveloped ambulance systems, and a lack of other transport options, delayed reaching care [19, 30, 35, 37, 53, 59, 63, 65, 77, 79, 90]. One of the studies reported that transportation fees further contribute to delays in reaching care (Table 2) [78].

### Factors associated with delay 3 (receiving care)

#### Delays in diagnosing and managing appendicitis

Forty-eight studies reported that diagnosing and managing appendicitis can be challenging (Table 2) [16, 18–26, 28, 32–35, 37–40, 44, 45, 47, 49, 50, 58, 59, 61, 65–68, 71–74, 76–80, 83–86, 88–90, 93]. Children with appendicitis may not be able to provide a detailed account of their symptoms [71, 90, 94], which along with an inadequate examination, and the irritability of children, may delay their diagnosis and management. Females with appendicitis may exhibit similar symptoms to several gynaecological diseases, leading to appendicitis being low on the index of suspicion [37, 38, 67]. One study reported that pregnant women preferred to consult their obstetrician first rather than a surgeon [86], and another found that both the obstetrician and surgeon were hesitant to operate on pregnant woman presenting with a query appendicitis [28]. HIV-positive patients also experience a delay in diagnosis and management when presenting with appendicitis [33].

#### Lack or shortage of human resources

Some of the studies cited a lack or shortage of experienced health-care staff as contributing to the delay in appendectomy [37, 39, 77]. High patient to staff ratios delayed prompt diagnosis [58]. Non-surgeon specialists working in smaller hospitals or emergency departments waited until symptoms worsened before referring patients [26, 38, 55, 58, 71].

### Lack of infrastructure

There is a lack of equipment and minimal funds to maintain the available equipment [22, 37, 47, 59, 72, 77, 88]. Limited theatre space at health-care facilities can also delay the administration of appendectomy [23, 55, 58]. Interfacility transfers can be delayed due to logistical issues such as insufficient bedspace at the referral hospital [69, 78], and transportation issues due to limited ambulance resources [35, 37, 65]. During COVID-19, delays to accessing appendectomy occurred due to a lack of surgical resources [31].

### Cultural and financial factors that affect receiving care

Seven studies reported that persons undergoing appendectomy may refuse to access care for reasons such as financial concerns [16, 18, 35, 37, 39, 55, 67], and obtaining permission from religious leaders prior to allowing the surgeon to proceed with the appendectomy.

#### Visualising the delays to accessing appendectomy

**Fig. 4:**
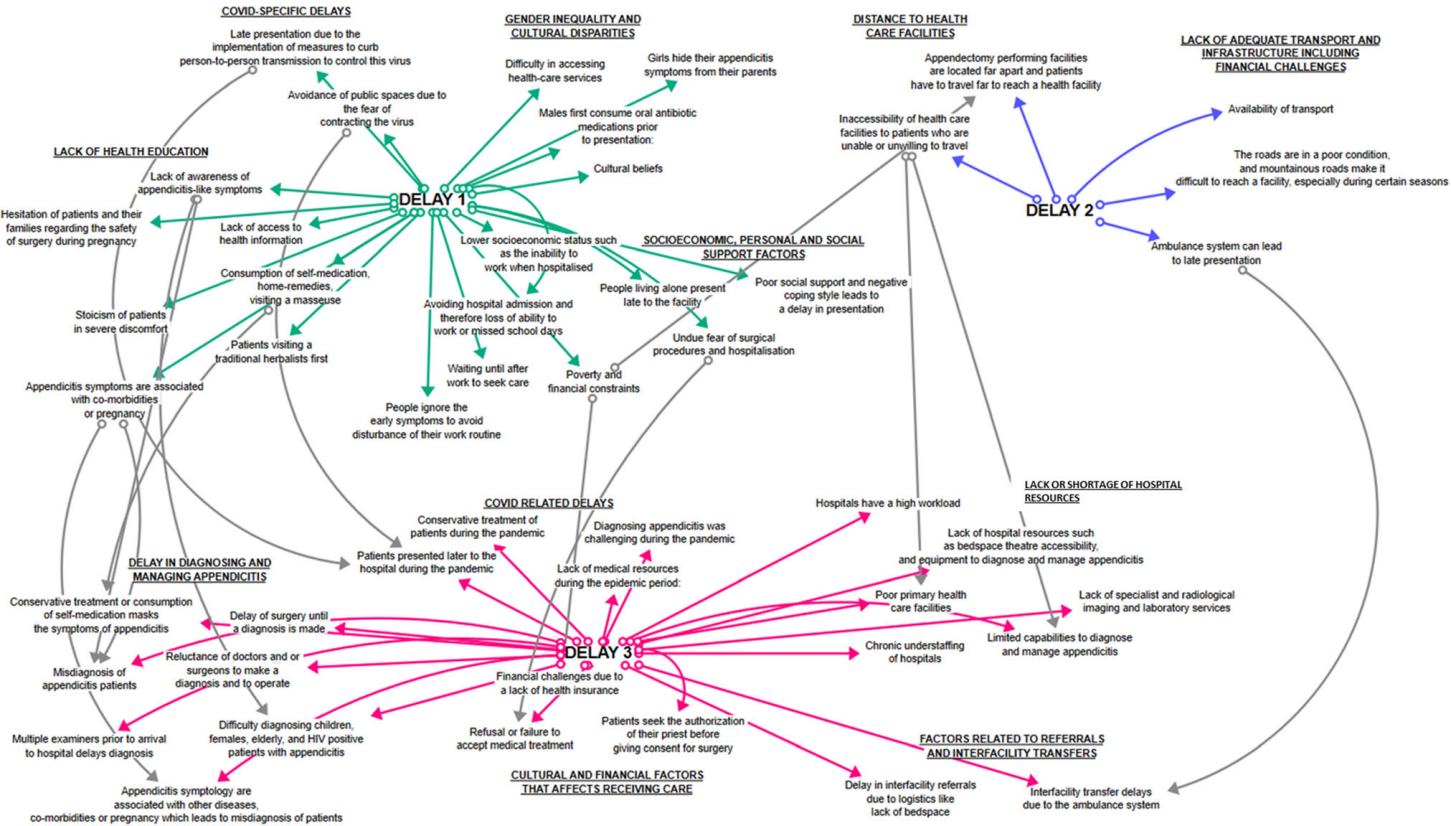
Interconnectivity of delays to appendectomy and their associated factors

Interconnectivities between factors and the associated delays that affected access to appendectomy were mapped (Fig. 4). For delays in seeking care, gender inequality was only associated with this delay. However, most of the factors grouped under a lack of health education (Delay 1—seeking care) could be linked to a delay in diagnosing and managing appendicitis (Delay 3—receiving care).

## Discussion

Our review identified and synthesised evidence from 78 studies on delays to appendectomy in LMICs [8]. All of the studies were quantitative [18, 20–28, 30–32, 34, 37, 39–42, 45, 46, 49–51, 57–60, 65–87, 89, 90]. Our review showed an uneven geographic distribution of studies from LMICs, with most publications emerging from South Africa, Nigeria, and Turkey [14, 15, 23, 24, 28, 29, 33, 35, 46, 50, 51, 53, 54, 60, 70, 81, 82, 84, 85, 90]. While findings from these countries may highlight important themes that can be extrapolated to other LMICs, they may not represent delays to appendectomy in all settings. More mixed methods and qualitative studies in LMICs, could add a richer dimension to understanding factors associated with delays in the appendectomy care pathway [95].

Overall, we acknowledge that there will be heterogeneity amongst countries in their social determinants of health, as well as their health systems, thus making any generalisations challenging. Nonetheless, our review included studies that reported morbidity and mortality rates among patients undergoing appendectomy. Patients may delay seeking care due to a lack of knowledge including the lack of perceived severity or urgency of appendicitis symptoms [96], and concerns relating to the perceived cost of health-care. Public awareness messaging should be targeted to specific populations with the greatest need for health education. Provision should also be made within the health system to enable access to free or affordable health-care [96].

The measured distance to a health-care facility and the cost of transportation both could be potential contributors to delays to care, although this was outside of the scope of the review. One determinant of equitable access to health-care is the ability to reach a facility that provides surgical care within two hours [1]. Few studies in our review documented that reaching appropriate surgical care was challenging, especially for rural communities. Similarly, a Pakistani study found that rural community dwellers were up to two times less likely to undergo an abdominal operation relative to urban dwellers [97]. Geographical access to health-care facilities and its effect on the administration of timely appendectomy needs to be addressed given the observed association between limited access to a health-care facility and high perforation rates [98, 99].

Barriers to receiving care in LMICs for appendectomy can be related to a lack of human and infrastructure resources. In sub-Saharan Africa there is a shortage in health-care workers [100, 101]. Trained health-care professionals are located within the city, leaving very few doctors available to help at district hospitals [97] which then forces individuals to travel to urban areas to obtain health-care [102]. Addressing the shortage of staff and trained providers could have a significant impact on the appendectomy care pathway if prioritised [103, 104]. Educational and ongoing training at lower-level facilities such as district hospitals are required. In some countries, decentralisation of certain procedures to alleviate the burden at higher-level facilities, and accessibility of affordable transport could aid in improving access to appendectomy.

For many individuals, multiple delays occur throughout the care pathway, such as financial barriers. Rural dwellers, especially, will delay seeking care owing to financial concerns, job insecurity, and a lack of access to a health-care facility [59]. Hence owing to the interconnectivity of barriers and delays, a health systems approach is needed to address these inequities. Engagement with policymakers and health system managers may be required to ensure that issues such as access due to socioeconomic factors, financial concerns, and geographical factors are considered when treatment protocols are being developed and implemented. This can be achieved by setting up stakeholder workshops, comprising of different role players, across countries. In the end these findings should be used to influence policy for appendectomy patients specifically.

This scoping review had limitations. Studies that were not in English and could not be translated using Google Translate, or no full-text article was available were excluded. Therefore, we may have underreported delays to accessing appendectomy. As distance and cost of care could not be studied because it was outside the scope of the review, its effect on time to accessing appendectomy could not be considered. The results are also limited in its generalisability due to the heterogeneity amongst countries in their social determinants of health and their health systems. However, to the best of our knowledge, this is the first study to synthesise evidence on delays to appendectomy in LMICs, using the Three Delays framework.

## Conclusion

This review has highlighted the need for additional studies on delays to accessing appendectomy in additional LMICs. While each health system is unique, there are some common themes. This review shows that in LMICs, persons seeking appendectomy present late to health-care facilities due to socioeconomic, cultural, and financial factors. After reaching a health-care facility, accessing appendectomy can further be delayed owing to a lack of resources that are required to provide the necessary care. Addressing these factors could improve patient outcomes. Future studies should also consider the heterogeneity of the health system within the relevant countries when interpreting these findings. Moreover, this work could inform a systematic review and meta-analysis that quantifies the impact of each of the delays so that it in turn better informs decision-making and budget allocation, improving access to appendectomy care.

### Supplementary Information

Below is the link to the electronic supplementary material.

Supplementary file 1 (DOCX 26 KB)

Supplementary file 2 (DOCX 79 KB)
