# Supplementary material for: Factors Contributing to Delays to Accessing Appendectomy in Low- and Middle-Income Countries: A Scoping Review
Source: World J Surg. 2023 Sep 25;47(12):3060–9. doi: 10.1007/s00268-023-07183-2 (PMC10694117; doi:10.1007/s00268-023-07183-2)
Supplement: Supplementary file 1 — (DOCX 26 KB) [file 268_2023_7183_MOESM1_ESM.docx]

**Supplementary Table 1. An example of our search strategy as used in the PubMed-Medline database**

**Note that the EPOC** **LMIC filters 2020 (v.4) using the following combination: #1b OR #2 were used to search for LMICs**

| #1 | appendicitis [MeSH Terms] |
| --- | --- |
| #2 | **appendectomy [MeSH Terms]** |
| #3 | **appendectomies [MeSH Terms]** |
| #4 | #1 OR #2 OR #3 |
| #5 | **appendi*[Title/Abstract]** |
| #6 | **appendec*[Title/Abstract]** |
| #7 | #5 OR #6 |
| #8 | **appendi*[Text Word]** |
| #9 | **appendec*[Text Word]** |
| #10 | #8 OR #9 |
| #11 | All fields: **appendec*** |
| #12 | All fields: **appendi*** |
| #13 | #11 OR #12 |
| #14 | #4 OR #7 OR #10 OR #13 |
| #15 | afghanistan**[Mesh:NoExp]** OR albania[Mesh:NoExp] OR algeria[Mesh:NoExp] OR american samoa[Mesh:NoExp] OR angola[Mesh:NoExp] OR argentina[Mesh:NoExp] OR armenia[Mesh:NoExp] OR azerbaijan[Mesh:NoExp] OR bangladesh[Mesh:NoExp] OR OR republic of belarus[Mesh:NoExp] OR belize[Mesh:NoExp] OR benin[Mesh:NoExp] OR bhutan[Mesh:NoExp] OR bolivia[Mesh:NoExp] OR bosnia and herzegovina[Mesh:NoExp] OR botswana[Mesh:NoExp] OR brazil[Mesh:NoExp] OR bulgaria[Mesh:NoExp] OR burkina faso[Mesh:NoExp] OR burundi[Mesh:NoExp] OR cabo verde[Mesh:NoExp] OR cambodia[Mesh:NoExp] OR cameroon[Mesh:NoExp] OR central african republic[Mesh:NoExp] OR chad[Mesh:NoExp] OR china[Mesh:NoExp] OR colombia[Mesh:NoExp] OR comoros[Mesh:NoExp] OR democratic republic of the congo[Mesh:NoExp] OR congo[Mesh:NoExp] OR costa rica[Mesh:NoExp] OR cote d’ivoire[Mesh:NoExp] OR cuba[Mesh:NoExp] OR djibouti[Mesh:NoExp] OR dominica[Mesh:NoExp] OR dominican republic[Mesh:NoExp] OR ecuador[Mesh:NoExp] OR egypt[Mesh:NoExp] OR el salvador[Mesh:NoExp] OR equatorial guinea[Mesh:NoExp] OR eritrea[Mesh:NoExp] OR swaziland[Mesh:NoExp] OR ethiopia[Mesh:NoExp] OR fiji[Mesh:NoExp] OR gabon[Mesh:NoExp] OR gambia[Mesh:NoExp] OR georgia (republic)[Mesh:NoExp] OR ghana[Mesh:NoExp] OR grenada[Mesh:NoExp] OR guatemala[Mesh:NoExp] OR guinea[Mesh:NoExp] OR guinea bissau[Mesh:NoExp] OR guyana[Mesh:NoExp] OR haiti[Mesh:NoExp] OR honduras[Mesh:NoExp] OR india[Mesh:NoExp] OR indonesia[Mesh:NoExp] OR iran[Mesh:NoExp] OR iraq[Mesh:NoExp] OR jamaica[Mesh:NoExp] OR jordan[Mesh:NoExp] OR kazakhstan[Mesh:NoExp] OR kenya[Mesh:NoExp] OR democratic people’s republic of korea[Mesh:NoExp] OR kosovo[Mesh:NoExp] OR kyrgyzstan[Mesh:NoExp] OR laos[Mesh:NoExp] OR lebanon[Mesh:NoExp] OR lesotho[Mesh:NoExp] OR liberia[Mesh:NoExp] OR libya[Mesh:NoExp] OR macau[Mesh:NoExp] OR republic of north macedonia[Mesh:NoExp] OR madagascar[Mesh:NoExp] OR malawi[Mesh:NoExp] OR malaysia[Mesh:NoExp] OR indian ocean islands[Mesh:NoExp] OR mali[Mesh:NoExp] OR micronesia[Mesh:NoExp] OR palau[Mesh:NoExp] OR mauritania[Mesh:NoExp] OR mauritius[Mesh:NoExp] OR mexico[Mesh:NoExp] OR moldova[Mesh:NoExp] OR mongolia[Mesh:NoExp] OR montenegro[Mesh:NoExp] OR morocco[Mesh:NoExp] OR mozambique[Mesh:NoExp] OR myanmar[Mesh:NoExp] OR namibia[Mesh:NoExp] OR nepal[Mesh:NoExp] OR nicaragua[Mesh:NoExp] OR niger[Mesh:NoExp] OR nigeria[Mesh:NoExp] OR pakistan[Mesh:NoExp] OR papua new guinea[Mesh:NoExp] OR paraguay[Mesh:NoExp] OR peru[Mesh:NoExp] OR philippines[Mesh:NoExp] OR russia[Mesh:NoExp] OR rwanda[Mesh:NoExp] OR samoa[Mesh:NoExp] OR sao tome and principe[Mesh:NoExp] OR senegal[Mesh:NoExp] OR serbia[Mesh:NoExp] OR sierra leone[Mesh:NoExp] OR melanesia[Mesh:NoExp] OR somalia[Mesh:NoExp] OR south africa[Mesh:NoExp] OR south sudan[Mesh:NoExp] OR sri lanka[Mesh:NoExp] OR saint lucia[Mesh:NoExp] OR saint vincent and the grenadines[Mesh:NoExp] OR sudan[Mesh:NoExp] OR suriname[Mesh:NoExp] OR syria[Mesh:NoExp] OR tajikistan[Mesh:NoExp] OR tanzania[Mesh:NoExp] OR thailand[Mesh:NoExp] OR timor leste[Mesh:NoExp] OR togo[Mesh:NoExp] OR tonga[Mesh:NoExp] OR trinidad and tobago[Mesh:NoExp] OR tunisia[Mesh:NoExp] OR turkey[Mesh:NoExp] OR turkmenistan[Mesh:NoExp] OR uganda[Mesh:NoExp] OR ukraine[Mesh:NoExp] OR uzbekistan[Mesh:NoExp] OR vanuatu[Mesh:NoExp] OR venezuela[Mesh:NoExp] OR vietnam[Mesh:NoExp] OR middle east[Mesh:NoExp] OR yemen[Mesh:NoExp] OR yugoslavia[Mesh:NoExp] OR zambia[Mesh:NoExp] OR zimbabwe[Mesh:NoExp] OR africa south of the sahara[Mesh:NoExp] OR africa, central[Mesh:NoExp] OR africa, northern[Mesh:NoExp] OR africa, southern[Mesh:NoExp] OR africa, eastern[Mesh:NoExp] OR africa, western[Mesh:NoExp] OR west indies[Mesh:NoExp] OR indian ocean islands[Mesh:NoExp] OR caribbean region[Mesh:NoExp] OR central america[Mesh:NoExp] OR latin america[Mesh:NoExp] OR south america[Mesh:NoExp] OR asia, central[Mesh:NoExp] OR asia, northern[Mesh:NoExp] OR asia, southeastern[Mesh:NoExp] OR asia, western[Mesh:NoExp] OR europe, eastern[Mesh:NoExp] OR developing countries[Mesh:NoExp] |
| #16 | Afghanistan **[Text Word]** OR albania[Text Word] OR algeria[Text Word] OR american samoa[Text Word] OR angola[Text Word] OR argentina[Text Word] OR armenia[Text Word] OR armenian[Text Word] OR azerbaijan[Text Word] OR bangladesh[Text Word] OR belarus[Text Word] OR byelarus[Text Word] OR belorussia[Text Word] OR byelorussian[Text Word] OR belize[Text Word] OR british honduras[Text Word] OR benin[Text Word] OR dahomey[Text Word] OR bhutan[Text Word] OR bolivia[Text Word] OR bosnia[Text Word] OR herzegovina[Text Word] OR botswana[Text Word] OR bechuanaland[Text Word] OR brazil[Text Word] OR brasil[Text Word] OR bulgaria[Text Word] OR burkina faso[Text Word] OR burkina fasso[Text Word] OR upper volta[Text Word] OR burundi[Text Word] OR urundi[Text Word] OR cabo verde[Text Word] OR cape verde[Text Word] OR cambodia[Text Word] OR kampuchea[Text Word] OR khmer republic[Text Word] OR cameroon[Text Word] OR cameron[Text Word] OR cameroun[Text Word] OR central african republic[Text Word] OR ubangi shari[Text Word] OR chad[Text Word] OR china[Text Word] OR colombia[Text Word] OR comoros[Text Word] OR comoro islands[Text Word] OR mayotte[Text Word] OR congo[Text Word] OR zaire[Text Word] OR costa rica[Text Word] OR cote d’ivoire[Text Word] OR cote d’ ivoire[Text Word] OR cote divoire[Text Word] OR cote d ivoire[Text Word] OR ivory coast[Text Word] OR cuba[Text Word] OR djibouti[Text Word] OR french somaliland[Text Word] OR dominica[Text Word] OR dominican republic[Text Word] OR ecuador[Text Word] OR egypt[Text Word] OR united arab republic[Text Word] OR el salvador[Text Word] OR equatorial guinea[Text Word] OR spanish guinea[Text Word] OR eritrea[Text Word] OR eswatini[Text Word] OR swaziland[Text Word] OR ethiopia[Text Word] OR fiji[Text Word] OR gabon[Text Word] OR gabonese republic[Text Word] OR gambia[Text Word] OR georgia[Text Word] OR georgian[Text Word] OR ghana[Text Word] OR gold coast[Text Word] OR grenada[Text Word] OR guatemala[Text Word] OR guinea[Text Word] OR guyana[Text Word] OR guiana[Text Word] OR haiti[Text Word] OR hispaniola[Text Word] OR honduras[Text Word] OR india[Text Word] OR indonesia[Text Word] OR timor[Text Word] OR iran[Text Word] OR iraq[Text Word] OR jamaica[Text Word] OR jordan[Text Word] OR kazakhstan[Text Word] OR kazakh[Text Word] OR kenya[Text Word] OR kosovo[Text Word] OR kyrgyzstan[Text Word] OR kirghizia[Text Word] OR kirgizstan[Text Word] OR kyrgyz republic[Text Word] OR kirghiz[Text Word] OR laos[Text Word] OR lao pdr[Text Word] OR lao people's democratic republic[Text Word] OR lebanon[Text Word] OR lesotho[Text Word] OR basutoland[Text Word] OR liberia[Text Word] OR libya[Text Word] OR libyan arab jamahiriya[Text Word] OR macau[Text Word] OR macedonia[Text Word] OR madagascar[Text Word] OR malagasy republic[Text Word] OR malawi[Text Word] OR nyasaland[Text Word] OR malaysia[Text Word] OR maldives[Text Word] OR indian ocean[Text Word] OR mali[Text Word] OR micronesia[Text Word] OR kiribati[Text Word] OR marshall islands[Text Word] OR palau[Text Word] OR tuvalu[Text Word] OR mauritania[Text Word] OR mauritius[Text Word] OR mexico[Text Word] OR moldova[Text Word] OR moldovian[Text Word] OR mongolia[Text Word] OR montenegro[Text Word] OR morocco[Text Word] OR ifni[Text Word] OR mozambique[Text Word] OR myanmar[Text Word] OR burma[Text Word] OR namibia[Text Word] OR nepal[Text Word] OR nicaragua[Text Word] OR niger[Text Word] OR nigeria[Text Word] OR muscat[Text Word] OR pakistan[Text Word] OR papua new guinea[Text Word] OR paraguay[Text Word] OR peru[Text Word] OR philippines[Text Word] OR philipines[Text Word] OR phillipines[Text Word] OR phillippines[Text Word] OR russia[Text Word] OR russian federation[Text Word] OR ussr[Text Word] OR soviet union[Text Word] OR union of soviet socialist republics[Text Word] OR rwanda[Text Word] OR ruanda[Text Word] OR samoa[Text Word] OR pacific islands[Text Word] OR polynesia[Text Word] OR samoan islands[Text Word] OR sao tome and principe[Text Word] OR senegal[Text Word] OR serbia[Text Word] OR sierra leone[Text Word] OR melanesia[Text Word] OR solomon island[Text Word] OR solomon islands[Text Word] OR norfolk island[Text Word] OR somalia[Text Word] OR south africa[Text Word] OR south sudan[Text Word] OR sri lanka[Text Word] OR ceylon[Text Word] OR saint lucia[Text Word] OR st lucia[Text Word] OR saint vincent[Text Word] OR st vincent[Text Word] OR grenadines[Text Word] OR sudan[Text Word] OR suriname[Text Word] OR surinam[Text Word] OR syria[Text Word] OR syrian arab republic[Text Word] OR tajikistan[Text Word] OR tadjikistan[Text Word] OR tadzhikistan[Text Word] OR tadzhik[Text Word] OR tanzania[Text Word] OR tanganyika[Text Word] OR thailand[Text Word] OR siam[Text Word] OR timor leste[Text Word] OR east timor[Text Word] OR togo[Text Word] OR togolese republic[Text Word] OR tonga[Text Word] OR trinidad[Text Word] OR tobago[Text Word] OR tunisia[Text Word] OR turkey[Text Word] OR turkmenistan[Text Word] OR turkmen[Text Word] OR uganda[Text Word] OR ukraine[Text Word] OR uzbekistan[Text Word] OR uzbek[Text Word] OR vanuatu[Text Word] OR new hebrides[Text Word] OR venezuela[Text Word] OR vietnam[Text Word] OR viet nam[Text Word] OR middle east[Text Word] OR west bank[Text Word] OR gaza[Text Word] OR palestine[Text Word] OR yemen[Text Word] OR yugoslavia[Text Word] OR zambia[Text Word] OR zimbabwe[Text Word] OR northern rhodesia[Text Word] OR global south[Text Word] OR africa south of the Sahara[Text Word] OR sub saharan Africa[Text Word] OR subsaharan africa[Text Word] OR central africa[Text Word] OR north africa[Text Word] OR northern africa[Text Word] OR magreb[Text Word] OR maghrib[Text Word] OR sahara[Text Word] OR southern africa[Text Word] OR east africa[Text Word] OR eastern africa[Text Word] OR west africa[Text Word] OR western africa[Text Word] OR west indies[Text Word] OR indian ocean islands[Text Word] OR caribbean[Text Word] OR central america[Text Word] OR latin america[Text Word] OR south america[Text Word] OR central asia[Text Word] OR north asia[Text Word] OR northern asia[Text Word] OR southeastern asia[Text Word] OR south eastern asia[Text Word] OR southeast asia[Text Word] OR south east asia[Text Word] OR western asia[Text Word] OR east europe[Text Word] OR eastern europe[Text Word] OR developing country[Text Word] OR developing countries[Text Word] OR developing nation[Text Word] OR developing nations[Text Word] OR developing population[Text Word] OR developing populations[Text Word] OR developing world[Text Word] OR less developed country[Text Word] OR less developed countries[Text Word] OR less developed nation[Text Word] OR less developed nations[Text Word] OR less developed world[Text Word] OR lesser developed countries[Text Word] OR lesser developed nations[Text Word] OR under developed country[Text Word] OR under developed countries[Text Word] OR under developed nations[Text Word] OR under developed world[Text Word] OR underdeveloped country[Text Word] OR underdeveloped countries[Text Word] OR underdeveloped nation[Text Word] OR underdeveloped nations[Text Word] OR underdeveloped population[Text Word] OR underdeveloped populations[Text Word] OR underdeveloped world[Text Word] OR middle income country[Text Word] OR middle income countries[Text Word] OR middle income nation[Text Word] OR middle income nations[Text Word] OR middle income population[Text Word] OR middle income populations[Text Word] OR low income country[Text Word] OR low income countries[Text Word] OR low income nation[Text Word] OR low income nations[Text Word] OR low income population[Text Word] OR low income populations[Text Word] OR lower income country[Text Word] OR lower income countries[Text Word] OR lower income nations[Text Word] OR lower income population[Text Word] OR lower income populations[Text Word] OR underserved countries[Text Word] OR underserved nations[Text Word] OR underserved population[Text Word] OR underserved populations[Text Word] OR underserved population[Text Word] OR underserved populations[Text Word] OR deprived countries[Text Word] OR deprived population[Text Word] OR deprived populations[Text Word] OR poor country[Text Word] OR poor countries[Text Word] OR poor nation[Text Word] OR poor nations[Text Word] OR poor population[Text Word] OR poor populations[Text Word] OR poor world[Text Word] OR poorer countries[Text Word] OR poorer nations[Text Word] OR poorer population[Text Word] OR poorer populations[Text Word] OR developing economy[Text Word] OR developing economies[Text Word] OR less developed economy[Text Word] OR less developed economies[Text Word] OR underdeveloped economies[Text Word] OR middle income economy[Text Word] OR middle income economies[Text Word] OR low income economy[Text Word] OR low income economies[Text Word] OR lower income economies[Text Word] OR low gdp[Text Word] OR low gnp[Text Word] OR low gross domestic[Text Word] OR low gross national[Text Word] OR lower gdp[Text Word] OR lower gross domestic[Text Word] OR lmic[Text Word] OR lmics[Text Word] OR third world[Text Word] OR lami country[Text Word] OR lami countries[Text Word] OR transitional country[Text Word] OR transitional countries[Text Word] OR emerging economies[Text Word] OR emerging nation[Text Word] OR emerging nations[Text Word] |
| #17 | #15 OR #16 |
| #10 | #14 AND #17 |

*Scopus, Web of Science, Africa Wide EBSCO host*: These databases were searched using keywords similar to those used in the search strategy for PubMed.

*African Journal Online (AJOL) and Bioline*: Keywords used in the search string applied in the above databases, were used in the search engines AJOL and Bioline.
