# Supplementary material for: Factors Contributing to Delays to Accessing Appendectomy in Low- and Middle-Income Countries: A Scoping Review
Source: World J Surg. 2023 Sep 25;47(12):3060–9. doi: 10.1007/s00268-023-07183-2 (PMC10694117; doi:10.1007/s00268-023-07183-2)
Supplement: Supplementary file 2 — (DOCX 79 KB) [file 268_2023_7183_MOESM2_ESM.docx]

***Supplementary Table 2*** *A summary of the included studies and the captured data*

| **Study Number** | **Author and year of publication** | **Low-and middle-income country** | **Study**  **Design** | **Delay**  **Assessed** | **Overarching theme** | **Relevant findings and reasons associated with the delay(s)** |
| --- | --- | --- | --- | --- | --- | --- |
| Africa |  |  |  |  |  |  |
|  | Afenigus AD, Bayieh AM, Kassahun B. 2020 | Ethiopia | Retrospective cross-sectional | 1,2 | - Delayed presentation to a health-care facility | Nearly a quarter of the patients developed unfavourable treatment outcomes of acute appendicitis possibly due to late presentation to the hospital. |
|  | Njoku TA, Okobia MN. 2006 | Benin | Retrospective cohort | 1,2 | - Delayed presentation to a health-care facility - Lack of knowledge of appendicitis symptoms and alternative forms of health-care - Financial concerns and personal factors | The main risk factors of perforated appendicitis in our study were delay in presenting to the hospital, lack of awareness of appendicitis-like symptoms, self-purgation, and comorbidities. |
|  | Bekele A, Mekasha A.  2006 | Ethiopia | Retrospective cross-sectional | 1,2,3 | - Delayed presentation to a health-care facility - Delay in diagnosing and managing appendicitis | The higher rate of perforations among under 10 year old children may be due to misdiagnosis and/or parental delay in bringing the child to a health-care facility. |
|  | Abantanga FA, Nimako B, Amoah M.  2009 | Ghana | Prospective cross-sectional survey | 1,2 | - Delayed presentation to a health-care facility - Lack of knowledge of appendicitis symptoms and alternative forms of health-care | The complication rate of appendicitis is increasing in children due to unawareness of appendicitis-like symptoms among parents, self-medication, and late presentation to a hospital. |
|  | Ohene-Yeboah M, Togbe B.  2006 | Ghana | Prospective cross-sectional | 1,2,3 | - Delayed presentation to a health-care facility - Delay in receiving care | Patients presented late as the mean duration of illness in our series was 74 hours and operations were delayed for 17 hours. |
|  | Willmore WS, Hill AG.  2001 | Kenya | Retrospective cohort | 2 | - Distance to health-care facilities | The facilities that can perform appendectomy are located far apart. Many patients must travel long distances to reach a health-care facility. |
|  | Langenscheidt P, Lang C, Pu¨schel W, *et al*.  1999 | Madagascar | Prospective cohort | 1,3 | - Gender and cultural disparities - Lack or shortage of human resources | Women more readily seek health-care in Madagascar than men for minor abdominal complaints. Hospitals have a high workload and are poorly staffed. |
|  | Williams BM, Purcell LN, Varela C, *et al.*  2021 | Malawi | Retrospective cross-sectional | 1,2,3 | - Delayed presentation to a health-care facility - Lack of infrastructure - Lack or shortage of human resources | Over 40% of the population presented late to the hospital. Most patients who died were transferred from an outside facility. The increased mortality may be due to the lack of a robust blood bank, insufficient critical care bed access, minimal ventilators, and limited intensive care unit expertise. This facility in Malawi also has a limited surgical and health-care workforce. |
|  | Agholor K, Omo-Aghoja L, Okonofua F.  2011 | Nigeria | Retrospective cross-sectional | 1,2,3 | - Lack of knowledge of appendicitis symptoms and alternative forms of health-care      - Distance to health-care facilities - Delay in diagnosing and managing appendicitis - Cultural and financial factors - Lack of infrastructure | Delays resulted as patients were either misdiagnosed, first sought pain-relief through a local masseuse, sought the authorization of their priest before giving consent for surgery, or experienced multiple interfacility referrals. |
|  | Adisa AO, Alatise OI, Arowolo OA, *et al.*  2012 | Nigeria | Prospective cohort | 1,3 | - Lack of knowledge of appendicitis symptoms and alternative forms of health-care - Delay in diagnosing and managing appendicitis - Lack of infrastructure | Patients used over the-counter drugs at the onset of symptoms and experienced a delay in referral from their primary care physicians. |
|  | Ahmed A, Dauda M, Garba S, *et al.*  2010 | Nigeria | Prospective cohort | 1,2,3 | - Delayed presentation to a health-care facility - Lack or shortage of human resources | Appendicitis patients presented late to health-care facilities. Lack of ultrasound facilities also makes it difficult to diagnose appendicitis early. |
|  | Ayoade BA, Olawoye OA, Salami BA, *et al.*  2006 | Nigeria | Retrospective cross-sectional | 1,3 | - Lack of knowledge of appendicitis symptoms and alternative forms of health-care - Financial concerns, and personal factors - Delay in diagnosing and managing appendicitis | Young, elderly, and female patients are challenging to diagnose. Patients presented late possibly due to the use of self-medication, and the fear of surgical procedures. The accuracy of ultrasound to diagnose appendicitis is operator dependent. Also, some of the subjects had socio-economic difficulties which necessitated delay in operative treatment. |
|  | Akhator A.  2012 | Nigeria | Retrospective cross-sectional | 3 | - Delay in diagnosing and managing appendicitis | Doctors are reluctant to make a diagnosis of appendicitis in the elderly which delays their treatment. |
|  | Duduyemi BM.  2015 | Nigeria | Retrospective cross-sectional | 1,2 | - Delayed presentation to a health-care facility | Late presentation to the emergency room led to an increase in complications in appendicitis patients. |
|  | Kunle AE, Yinka OT, Oluyomi TA, *et al.*  2011 | Nigeria | Retrospective cross-sectional | 1,3 | - Lack of knowledge of appendicitis symptoms and alternative forms of health-care - Financial concerns, and personal factors - Delay in diagnosing and managing appendicitis - Lack or shortage of human resources - Cultural and financial factors | Some patients choose to visit traditional herbalists before visiting a formal health-care facility due to financial challenges. Inexperienced personnel tend to misdiagnose patients. These factors lead to late presentation and a delay in treatment, respectively. Patients also pay directly for their treatment due to a lack of health insurance which leads to in-hospital delays. |
|  | Shambe IH, Dikkol N, Ozoilo KN.  2016 | Nigeria | Retrospective cross-sectional | 1 | - Lack of knowledge of appendicitis symptoms and alternative forms of health-care | Most women may confuse the symptoms of appendicitis with the normal symptoms of pregnancy. This may lead to a delay in presentation. |
|  | Osuigwe AN, Ekwunife CN, Anyanwu SNC.  2004 | Nigeria | Retrospective cohort | 1,2,3 | - Delayed presentation to a health-care facility - Delay in diagnosing and managing appendicitis | The high rate of complicated appendicitis in our children is related to prolonged pre-hospital delay due to a low index of suspicion or a flaw in our diagnostic methods. |
|  | Ademola TO, Oludayo SA, Samuel OA, *et al.*  2015 | Nigeria | Retrospective cohort | 1,2 | - Delayed presentation to a health-care facility | There was a statistically significant relationship between the duration of the disease at presentation and the likelihood of the disease being complicated. |
|  | Edino ST, Mohammed AZ, Ochicha O, *et al.*  2004 | Nigeria | Retrospective cohort | 1,2,3 | - Delayed presentation to a health-care facility - Delay in diagnosing and managing appendicitis - Cultural and financial factors | Majority of the patients used antibiotics from peripheral hospitals prior to presentation. Delayed presentation, fulminant disease, misdiagnosis, or failure to accept medical treatment, are contributory factors to high perforation rates. |
|  | Osifo OD, Ogiemwonyi SO.  2009 | Nigeria | Retrospective cohort | 1,2,3 | - Delayed presentation to a health-care facility - Lack of knowledge of appendicitis symptoms and alternative forms of health-care - Financial concerns, and personal factors - Delay in diagnosing and managing appendicitis - Lack of infrastructure | Fifty-two patients were referred late after wrong diagnoses and treatment with different complications. Delayed referrals and complications were more among children living in rural areas who did not seek medical attention early due mainly to ignorance and financial constraints. The clinical diagnosis of appendicitis is difficult in children because of their inability to give an accurate history, many differentials, lack of cooperation during examination and the absence of classical signs and symptoms. This may be compounded by medications taken before presentation that may have altered the clinical picture as shown in this study. |
|  | Hernandez MC, Kong VY, Bruce JL, *et al.*  2018 | South Africa | Retrospective cohort | 1,2 | - Delayed presentation to a health-care facility | A delay in presentation to a hospital leads to postoperative complications. |
|  | Kong VY, Aldous C, Clarke DL.  2014 | South Africa | Prospective cross-sectional | 1,2,3 | - Delayed presentation to a health-care facility - Delay in diagnosing and managing appendicitis - Lack of infrastructure | Patients were inappropriately discharged from the hospital and admitted prior to referral for definitive surgical care. Ten percent of patients used traditional medicine and most of the patients presented late. Appendicitis patients are at risk for delayed diagnosis after entering the health system. |
|  | Kong VY, Bulajic B, Allorto NL, *et al.*  2012 | South Africa | Prospective cross-sectional | 1,2,3 | - Delayed presentation to a health-care facility - Gender and cultural disparities - Distance to health-care facilities - Lack of adequate transport and transport infrastructure - Lack of infrastructure | Delays to definitive therapy leads to perforation and a longer length of hospital stay. The reasons for late presentation include cultural factors and difficulty in accessing health-care services. Referrals from the rural hospitals made up 35% of all admissions. |
|  | Kong VY, van de Linde S, Aldous C, *et al.*  2013 | South Africa | Prospective cohort | 1,2,3 | - Delayed presentation to a health-care facility - Lack of knowledge of appendicitis symptoms and alternative forms of health-care - Financial concerns, and personal factors - Distance to health-care facilities - Lack of adequate transport and transport infrastructure - Delay in diagnosing and managing appendicitis - Lack or shortage of human resources - Lack of infrastructure | Rural patients had a longer delay in presentation than urban patients. Parental or patient recognition of the potential urgency of the illness and timely health-seeking behaviour followed by clinical recognition of potential appendicitis, appropriate referral, and surgical intervention are crucial. Rural areas experience chronic understaffing of hospitals and high staff turnover, and lack specialist and radiological imaging and laboratory services. The possibility of hospital admission and therefore loss of ability to work or missed school days potentially prevented many patients from seeking medical attention. Accessing clinics in KwaZulu-Natal requires substantial travelling. All patients with appendicitis are referred to the regional hospital for further assessment, thus creating two further potential delays – in diagnosis/recognition and in interfacility transportation for surgery. |
|  | Kong VY, Sartorius B, Clarke DL  2015 | South Africa | Retrospective cross-sectional | 1,2,3 | - Delayed presentation to a health-care facility - Distance to health-care facilities - Delay in diagnosing and managing appendicitis - Lack or shortage of human resources - Lack of infrastructure - Cultural and financial factors | Most patients from the rural regions have a delay in presentation. Health-care facilities can be inaccessible due to the distance from the rural hospital facilities. Delays are also encountered when patients are assessed at rural hospitals, as the experience of staff at these facilities differs. Interfacility transfers are associated with logistical difficulties. Diagnosing female patients is especially challenging. The financial implications of inadequate and poor-quality surgical services are stark. |
|  | Nshuti R, Kruger D, Luvhengo TE  2014 | South Africa | Prospective cross-sectional | 1,2,3 | - Lack of knowledge of appendicitis symptoms and alternative forms of health-care - Distance to health-care facilities - Lack of adequate transport and transport infrastructure - Delay in diagnosing and managing appendicitis - Lack of infrastructure | The most common reasons for delay in presentation were a lack of access to hospitals or clinics and to information, a lack of disease awareness and health-care facilities, self-medication, prior treatment by a doctor, lack of access, a delay in transfer, and differential diagnosis. According to this study elderly patients with appendicitis have an atypical clinical presentation, most often with associated co-morbidities. |
|  | Levy RD, Degiannis E, Kantarovsky A, *et al.*  1997 | South Africa | Prospective audit (cross sectional) | 1,2,3 | - Delayed presentation to a health-care facility - Lack of knowledge of appendicitis symptoms and alternative forms of health-care - Distance to health-care facilities - Lack of adequate transport and transport infrastructure - Delay in diagnosing and managing appendicitis | The relatively high perforation rates in this study reflect patients’ attitude to illness, lack of education, late presentation and poor access to medical care. It can also be difficult for providers to differentiate appendicitis from gynaecological conditions. |
|  | Moustakis J, Piperidis AA, Ogunrombi AB  2020 | South Africa | Retrospective cross-sectional | 1,2 | - Delayed presentation to a health-care facility | We observed a decrease in time from admission to appendicectomy, probably owing to fewer patients utilising surgical services, and so reducing theatre waiting times. |
|  | Fulton J,  Lazarus C.  1995 | South Africa | Prospective cross-sectional | 1,2,3 | - Lack of knowledge of appendicitis symptoms and alternative forms of health-care - Financial concerns, and personal factors - Lack of adequate transport and transport infrastructure - Lack or shortage of human resources | Poor primary health-care facilities, lack of patient education, and poverty continue to lead to a delay to accessing care. The stoicism of many patients in severe discomfort and the lack of adequate transport may have added to the delay in presentation. |
|  | Sobnach S, Ede C , Van Der Linde G, *et al.*  2019 | South Africa | Retrospective cohort | 1,2 | - Delayed presentation to a health-care facility - Distance to health-care facilities - Lack of adequate transport and transport infrastructure | Many of our patients come from rural areas and access to health-care facilities can be difficult due to the geography of the Northern Cape province. Delayed presentation to hospital was more common in the HIV positive patient cohort. |
|  | Rogers Ad, Hampton Mi, Bunting M, *et al.*  2008 | South Africa | Retrospective cohort | 1,2,3 | - Delayed presentation to a health-care facility - Lack of knowledge of appendicitis symptoms and alternative forms of health-care - Distance to health-care facilities - Lack of adequate transport and transport infrastructure - Delay in diagnosing and managing appendicitis - Lack of infrastructure - Lack or shortage of human resources | In older patients, delayed presentation and a decreased index of suspicion for appendicitis could lead to increased perforation rates. Patient ignorance, the use of traditional medicine, poor access to medical services, inadequately staffed referral centres, and the ambulance system, can also add to their late presentation. |
|  | Kanumba ES, Mabula JB, Rambau P, *et al.*  2011 | Tanzania | Prospective cross-sectional | 1,2,3 | - Financial concerns, and personal factors - Lack of adequate transport and transport infrastructure - Delay in diagnosing and managing appendicitis - Lack of infrastructure - Cultural and financial factors | The reasons for delay in seeking medical consultation in this study may be due to interfacility transfer delays, financial and transport challenges, or misdiagnosis. Patients who fear surgery are treated conservatively with analgesics and antibiotics which then mask the symptoms making the diagnosis of appendicitis challenging. |
|  | Giiti GC, Mazigo HD, Heukelbach J, *et al.*  2010 | Tanzania | Prospective cross-sectional | 3 | - Delay in diagnosing and managing appendicitis | HIV-positive patients experience a delay in diagnosis and thus a delay in surgical interventions. |
| America |  |  |  |  |  |  |
|  | Fonseca MK, Trindade EN, Filho OPC, *et al.*  2020 | Brazil | Retrospective cross-sectional | 1,2 | - Delayed presentation to a health-care facility - Gender and cultural disparities | Patients presented late as they avoided health-care facilities due to the fear of contracting the virus. This led to a decrease in urgent surgical activity during the pandemic. Rigorous social distancing policies were implemented by governments and health entities worldwide, encouraging people to stay at home and avoid visiting local medical facilities. |
|  | Steinman M, Rogeri PS, Lenci LL, *et al.*  2013 | Brazil | Retrospective cross-sectional | 1,2,3 | - Delayed presentation to a health-care facility - Financial concerns, and personal factors - Gender and cultural disparities - Delay in receiving care - Delay in diagnosing and managing appendicitis | Patients from the public hospital wait longer for surgery and have a longer length of stay compared to private hospital patients. This difference may be caused by underlying socioeconomic and cultural disparities that might influence a delayed decision to be seen by a doctor. The time it takes to complete preoperative exams is also longer in the public health system. |
| Eastern Mediterranean |  |  |  |  |  |  |
|  | Magaam S.  2011 | Egypt | Intervention study - non-randomised control trial | 2,3 | - Lack of adequate transport and transport infrastructure - Delay in diagnosing and managing appendicitis - Lack or shortage of human resources | Young children with appendicitis are challenging to diagnose as it is difficult to obtain their history and determine their clinical signs. In our study, complications were due to delayed presentation of the patients (especially from rural areas) into the hospital due to difficulties of traffic on the mountainous roads which delay the diagnosis and treatment of the disease. With laparoscopic appendectomy limitations can include technical difficulty and unavailability of the equipment. |
|  | Seqsaqa M, Rozeik AE, Khalifa M, *et al.*  2021 | Egypt | Prospective cohort (comparative study) | 1,2 | - Lack of knowledge of appendicitis symptoms and alternative forms of health-care - Financial concerns, and personal factors - Distance to health-care facilities - Lack of adequate transport and transport infrastructure | Rural areas have lower education, health awareness, and socioeconomic levels, in addition to difficulties in transportation facilities. Geographic status affects the postoperative outcome in children with complicated appendicitis. Children have difficulties in understanding and expressing their complaints, and therefore more commonly present with perforation. |
|  | Sayyahfar S, Nasiri J, Dehghan KT, *et al.*  2019 | Iran | Retrospective observational case series | 1,3 | - Lack of knowledge of appendicitis symptoms and alternative forms of health-care - Delay in diagnosing and managing appendicitis | Fifteen percent of patients were first misdiagnosed in other centres, before being referred. Younger children have a high rate of misdiagnosis due to the inability to provide a detailed clinical history and describe all the red flags. |
|  | Paydar S, Parsijani PJ, Akbarzadeh A, *et al.*  2013 | Iran | Retrospective cross-sectional | 3 | - Delay in diagnosing and managing appendicitis - Lack or shortage of human resources | There was a high rate of misdiagnosed appendicitis in this study possibly due to the inappropriate use of available diagnostic devices, physicians' diagnostic skills, and the high workload of the emergency department of the hospital. |
|  | Mohammed IF, AlBayati BA.  2020 | Iraq | Prospective cohort | 1,2 | - Delayed presentation to a health-care facility | Patients who presented late had appendicular perforation with purulent peritonitis on diagnosis. |
|  | Duqoum W.  2001 | Jordan | Retrospective cross-sectional | 3 | - Delay in diagnosing and managing appendicitis | The higher rate of perforation could be explained by the delay in diagnosis as the clinical picture of appendicitis may overlap with diseases of pregnancy itself. Both the surgeon and the obstetrician were also hesitant to subject a pregnant woman to anaesthesia and surgery, which led to a delayed diagnosis. |
|  | Asad S, Ahmed A, Ahmad S, *et al.*  2015 | Pakistan | Prospective cross-sectional | 1,2,3 | - Lack of knowledge of appendicitis symptoms and alternative forms of health-care - Financial concerns, and personal factors - Lack of adequate transport and transport infrastructure - Delay in diagnosing and managing appendicitis | Due to a lack of proper roads and transportation facilities in most of the suburban and rural areas, it takes longer to reach the hospital. In the rural areas, people are preoccupied with their busy routine and they ignore the early symptoms to avoid disturbance of their work. The nature of the health-care system in Pakistan is such that most of the acute appendicitis patients are first checked by the general practitioner, medical technician, quacks etc. Some of the patients were initially misdiagnosed and sent home as an outpatient case. Others were managed conservatively or were delayed at home and did not take any consultation. Patients who are treated with antibiotics symptoms are masked and they become pain-free and no longer seek medical attention. |
|  | Fahim F, Shirjeel S.  2005 | Pakistan | Prospective cohort | 3 | - Delay in diagnosing and managing appendicitis - Lack of infrastructure - Lack or shortage of human resources | A longer average delay period was seen in misdiagnosed cases. In-hospital logistics like heavy workload and non-availability of operating theatres may cause a further unavoidable delay. |
|  | Khan MS, Siddiqui MTH, Shahzad N, *et al.*  2019 | Pakistan | Retrospective case-control | 1,2 | - Delayed presentation to a health-care facility | Patients in the complicated appendicitis group had symptoms for longer and experienced more complications. |
|  | AHMAD N, ALI AA.  2020 | Pakistan | Prospective  case series | 1,2,3 | - Delayed presentation to a health-care facility - Financial concerns, and personal factors - Delay in diagnosing and managing appendicitis | Poverty, late clinical presentation, misdiagnosis, elderly age, and a lack of modern diagnostic modalities were the major factors responsible for high morbidity and longer hospital stay. Most of the patients belonged to the poor class and were treated repetitively by the local doctors. |
|  | Rana MUI, Amna A, Fawad M.  2018 | Pakistan | Retrospective cohort | 1,2,3 | - Delayed presentation to a health-care facility - Delay in diagnosing and managing appendicitis | Medication can change the signs and complications of appendicitis making the disease difficult to diagnose. Late presentation leads to an increase in complications. |
|  | Ghumro AA, Khaskheli NM, Memon AA, *et al.*  1996 | Pakistan | Prospective cross sectional | 1,2,3 | - Delayed presentation to a health-care facility - Lack of knowledge of appendicitis symptoms and alternative forms of health-care - Delay in diagnosing and managing appendicitis - Lack of infrastructure | The increased perforation incidence is probably due to improper health-care facilities and referral systems due to quackery and ignorance about the disease, use of herbal medicines and the reluctance of the patient for surgery, and a delay in administering the operation. |
|  | Azoz MEH, Elhaj MA.  2009 | Sudan | Retrospective audit (cross-sectional) | 1,3 | - Lack of knowledge of appendicitis symptoms and alternative forms of health-care - Delay in diagnosing and managing appendicitis | Irritability of the child, the inability of proper communication with parents, atypical presentation, and the administration of antispasmodic or analgesic prior to presentation may delay the diagnosis of appendicitis. |
|  | Doumi EAB, Abdelrahman IH.  2007 | Sudan | Retrospective audit (cross-sectional) | 3 | - Delay in diagnosing and managing appendicitis - Lack of infrastructure | All our patients were misdiagnosed and thus incorrectly treated. These findings reflect the poor awareness of acute appendicitis and its seriousness in this area. |
|  | Doumi EBA, Mohammed MI.  2009 | Sudan | Prospective cross-sectional study | 1,3 | - Lack of knowledge of appendicitis symptoms and alternative forms of health-care - Delay in diagnosing and managing appendicitis | Many patients presented late due to poor awareness about the condition and its seriousness among the people, and easy access to self-medication. Patients were also frequently misdiagnosed at primary health-care settings. |
|  | Doumi EA and Manofali OE.  2014 | Sudan | Prospective cohort | 1,2,3 | - Delayed presentation to a health-care facility - Delay in diagnosing and managing appendicitis - Lack or shortage of human resources | Most of the patients were from rural areas and presented late. These patients then also presented to the emergency room when only junior staff with limited surgical experience and little backup in the field of general anaesthesia or intensive care units were available. In our study many patients first visited a rural primary health setting (medical assistant or nurse) but due to the poor awareness about acute appendicitis and its seriousness among the health providers in this area, the diagnosis was missed or the patients were mismanaged. |
|  | Ghali MAE, Kaabia O, Mefteh ZB, *et al.*  2018 | Tunisia | Retrospective case series | 3 | - Delay in diagnosing and managing appendicitis | Pregnant women with appendicitis experience a delay in diagnosis and/or in their management which leads to complications. |
|  | Miloudi N, Brahem M, Ben Abid S, *et al.*  2012 | Tunisia | Retrospective case series | 1,2,3 | - Delayed presentation to a health-care facility - Delay in diagnosing and managing appendicitis | Diagnosing appendicitis in pregnancy is complicated. Certain clinical signs may be misconstrued as pregnancy-related symptoms. |
| Europe |  |  |  |  |  |  |
|  | Calis H  2018 | Turkey | Retrospective cohort | 1,2,3 | - Delayed presentation to a health-care facility - Delay in diagnosing and managing appendicitis | Patients presented late to the health-care facility. Also diagnosing elderly patients is challenging. |
|  | Gurleyik G & Gurleyik E | Turkey | Retrospective cross-sectional | 3 | - Delay in diagnosing and managing appendicitis | The diagnosis of appendicitis in geriatric patients is occasionally difficult because of atypical and sometimes misleading physical findings. Doctors are also hesitant to make a diagnosis of appendicitis in the elderly patients. |
|  | Ibis C, Albayrak D, Hatipoglu AR,  *et al.*  2010 | Turkey | Retrospective cohort | 1,2,3 | - Delayed presentation to a health-care facility - Lack or shortage of human resources - Lack of infrastructure | Elderly patients tend to present late to the hospital. Also, the limited intensive care bedspace in our region delays interfacility referrals of patients to a tertiary hospital prior to surgery. |
|  | Ozguner IF, Buyukyavuz BI, Savas MC.  2004 | Turkey | Retrospective cohort | 1,2,3 | - Delayed presentation to a health-care facility - Lack of knowledge of appendicitis symptoms and alternative forms of health-care - Financial concerns, and personal factors - Delay in diagnosing and managing appendicitis | In our patients, drug usage before admission was seen in 55% of perforated patients. In all of these patients’ medication was started by a general practitioner or paediatricians, which frequently led to misdiagnosis. Patients may not be able to provide a good history of their symptoms, and the aetiology of abdominal pain is often unclear. In uncertain cases, a delay of surgery and close follow up in hospitals are common to reach a more precise diagnosis, which can lead to complications. However, parental delay was due to the low socioeconomic status in our area. |
|  | Terzi A, Yildiz F, Vural M.  2010 | Turkey | Retrospective case series | 3 | - Delay in diagnosing and managing appendicitis | Diagnosis of appendicitis is challenging due to pain and other symptoms that may be caused by pregnancy. There is also a decreased willingness to operate during pregnancy. Pregnant patients are usually first examined by obstetricians or by emergency room physicians. One reason for a delay in diagnosis is late examination and therefore delayed hospital admission by the general surgery specialist. |
|  | Sulu B, Gunerhan Y, Palanci Y, *et al.*  2010 | Turkey | Retrospective cohort | 1,2,3 | - Lack of knowledge of appendicitis symptoms and alternative forms of health-care - Lack of adequate transport and transport infrastructure - Delay in receiving care | Patients present late to the hospital, due to the high rate of rural residences around Kars together with difficulties in communication and transportation. |
|  | Turanli S, Kiziltan G.  2021 | Turkey | Retrospective cohort | 1,2,3 | - Delayed presentation to a health-care facility - Financial and personal factors - Delay in diagnosing and managing appendicitis | During the pandemic there was an increase in perforation rates due to the fear of contracting COVID-19 in public spaces such as hospitals. During the pandemic, the perforation rate increased. The diagnosis of appendicitis may be challenging during the COVID-19 pandemic. During the pandemic period, patients were given conservative treatment to keep their hospitalization time to a minimum. |
| Multiple countries |  |  |  |  |  |  |
|  | Reiter AJ, Schlottmann F, Kajombo C.  2019 | Argentina, Malawi | Retrospective cohort | 1,2,3 | - Delayed presentation to a health-care facility - Lack of knowledge of appendicitis symptoms and alternative forms of health-care - Financial concerns, and personal factors - Gender and cultural disparities      - Distance to health-care facilities - Lack of adequate transport and transport infrastructure - Lack of infrastructure - Delay in diagnosing and managing appendicitis | In most countries in sub-Saharan Africa, surgical care is usually concentrated in central urban tertiary hospitals that are inaccessible to patients who are unable or unwilling to travel. Health-seeking behaviours are also influenced by gender inequalities, educational attainment, awareness, and cultural beliefs. There is also a difference in the health-care workforce distribution, theatre availability, and hospital efficiency and organization. In low-income countries, the experience of medical and surgical staff is heterogeneous. This leads to diagnostic uncertainty, inappropriate periods of observation, and/or inappropriate discharge of the patients. The classical clinical features may only be present in a third of the patients in Malawi. Therefore, the clinical diagnosis may be more challenging in these populations. |
|  | Hernandez MC, Finnesgaard E, Aho JM, *et al.*  2018 | South Africa | Retrospective cohort | 1,2 | - Delayed presentation to a health-care facility | Rural patients presented later to the hospital than the urban patients which led to complicated appendicitis. |
|  | Avci V, Ayengin K.  2019 | Turkey | Retrospective cross-sectional | 1 | - Lack of knowledge of appendicitis symptoms and alternative forms of health-care - Gender and cultural disparities | Girls were taken to the hospital later than boys due to inadequate education of girls living in our region or the fact that families do not prioritize girls as much as boys. In rural areas particularly, girls’ shyness may lead to them hiding their symptoms from their parents, resulting in delayed presentation to health-care institutions. |
| South-east Asia |  |  |  |  |  |  |
|  | Rahman M, Chowdhury TK, Chowdhury MZ, *et al.*  2020 | Bangladesh | Retrospective cohort | 1,2,3 | - Delayed presentation to a health-care facility - Lack of knowledge of appendicitis symptoms and alternative forms of health-care - Lack or shortage of human resources - Lack of infrastructure | Delays in presentation was commonly seen in this study. These patients already tried some oral or parenteral antibiotics prescribed by a rural doctor or a physician. Laboratory tests were only done for 40% of the patients to prevent additional delays. There was a delay in diagnosis due to limited availability of operating theatres. Young children with suspected appendicitis need to be referred and managed early. |
|  | De U, Ghosh S.  2002 | India | Prospective  case series | 1 | - Financial concerns, and personal factors | Patients from the lower socioeconomic groups avoided operation until it was necessary fearing operative expenditure and the loss of working time. |
|  | Maroju NK, Smile SR, Sistla SC, *et al.*  2004 | India | Prospective cohort | 1,2,3 | - Delayed presentation to a health-care facility - Lack of knowledge of appendicitis symptoms and alternative forms of health-care - Financial concerns, and personal factors - Distance to health-care facilities - Lack of adequate transport and transport infrastructure - Lack of infrastructure - Cultural and financial factors | Delays in accessing appendectomy resulted from a lack of a relative with the patient or limited theatre availability. The results of the present study indicate that patient factors are the most important determinants influencing stage of the appendicitis at which surgery takes place. Late presentation leads to complications, which can also be caused by patients not being educated to a high school level, financial challenges, and living in suburban areas where medical care is difficult to access. |
|  | Singh M, Kadian YS, Rattan KN, *et al.*  2014 | India | Prospective cross-sectional | 1,2,3 | - Lack of knowledge of appendicitis symptoms and alternative forms of health-care - Distance to health-care facilities - Lack of adequate transport and transport infrastructure - Delay in diagnosing and managing appendicitis | Diagnosing children can be challenging which can lead to delays due to inadequate communication regarding their symptoms. Patients who do not have good access to medical care are more likely to present with perforation. Patients from rural areas have higher rates of perforation with appendicitis. |
|  | Bhandari TR, Shahi S, Acharya S.  2017 | Nepal | Retrospective cohort | 1,2,3 | - Delayed presentation to a health-care facility - Lack of knowledge of appendicitis symptoms and alternative forms of health-care - Financial concerns, and personal factors - Lack of adequate transport and transport infrastructure - Distance to health-care facilities - Delay in diagnosing and managing appendicitis - Lack or shortage of human resources | Our patients presented very late to the hospital thus delaying timely diagnosis and treatment. Challenges faced at the facility include scarcity of trained manpower and training opportunities, frequent usage of disposable surgical tools, shortage of funds to preserve equipment, and poor postoperative care. Access to medical services is limited due to the poor economy, poor transportation services, and long distances in rural setting. Mostly. our patients and their families hesitate regarding the safety of surgery during pregnancy and do not realize the advantage due to lack of medical knowledge, poor education, and social stigma. |
|  | Gupta A, Regmi S, Hazra NK, *et al.*  2010 | Nepal | Retrospective cohort | 1,2,3 | - Delayed presentation to a health-care facility - Delay in diagnosing and managing appendicitis | Patients had a delay in presentation and/or a delay in surgery. |
|  | Pokharel N, Sapkota P, KC B, *et al.*  2011 | Nepal | Retrospective cohort | 1,3 | - Lack of knowledge of appendicitis symptoms and alternative forms of health-care - Financial concerns, and personal factors - Delay in diagnosing and managing appendicitis | Co-morbidities frequently imply that symptomatology for appendicitis may be confused with already existing symptoms making the clinical diagnosis of elderly patients difficult. Also, concurrent medication may further complicate this issue. The elderly patient frequently refuses medical care and this can further impede appropriate management. |
|  | Bagguley D, Fordyce A, Guterres J, *et al.*  2019 | Timor Leste | Prospective cross-sectional study | 1,2,3 | - Lack of knowledge of appendicitis symptoms and alternative forms of health-care - Distance to health-care facilities - Delay in diagnosing and managing appendicitis - Lack or shortage of human resources | Patients arrived at the hospital via ambulance, private transportation (car/ motorbike), taxi, walking, and airplane. There was a delay in seeking care due to the use of traditional medicine before presentation to a community health clinic or hospital. The greatest delay was the first delay stage for emergency laparotomies. Delays in receiving care resulted from inadequate fasting status, theatre access, awaiting surgical review, awaiting imaging results, and preoperative blood transfusion. In Timor Leste there is a delay in patients deciding their condition needs hospital treatment when they have acute abdominal pain. |
| Western Pacific |  |  |  |  |  |  |
|  | Gao Z, Li M, Zhou H, *et al.*  2020 | China | Retrospective cross-sectional | 1,2,3 | - Delayed presentation to a health-care facility - Lack of knowledge of appendicitis symptoms and alternative forms of health-care - Financial concerns, and personal factors - Delay in receiving care - Lack of infrastructure | In the epidemic group, patients presented later and more patients asked for non-surgical treatment strategies after the outbreak as compared with the cases before the outbreak. There may also have been a shortage of medical resources during the epidemic period. Many patients chose to self-medicate or received antibiotic therapy in outpatient or private clinics before being hospitalized for further treatment. Measures to curb person-to-person transmission which were implemented to control this virus affected health-seeking behaviour. |
|  | Li J, Xu R, Hu D, *et al.*  2019 | China | Prospective cross-sectional | 1,2 | - Delayed presentation to a health-care facility - Lack of knowledge of appendicitis symptoms and alternative forms of health-care - Financial concerns, and personal factors | Delays were experienced by patients who were aged ≥60 years and living alone. A history of appendicitis among acquaintances may encourage patients to seek medical help when suffering from abdominal pain. The symptoms of patients in the delay group were more likely to have occurred on a working day. Patients’ poor social support and negative coping style were significantly associated with prehospital delay. |
|  | Zhou Y & Cen LS.  2020 | China | Retrospective cohort | 1,2,3 | - Delayed presentation to a health-care facility - Lack of knowledge of appendicitis symptoms and alternative forms of health-care - Delay in diagnosing and managing appendicitis | The chief complaint duration for perforated appendicitis patients in 2020 was longer than that for 2019. The emergency preoperative assessment time was longer in 2020 than in 2019. Some patients with mild symptoms may have achieved relief by taking pills themselves. There was also a delay in diagnosing and treating appendicitis. |
|  | Ahmad KA, Ideris N, Aziz SHSA.  2019 | Malaysia | Retrospective cross-sectional | 1 | - Lack of knowledge of appendicitis symptoms and alternative forms of health-care - Gender and cultural disparities | Males may have a higher threshold for pain compared to females, and most of them had a history of oral antibiotic medications. Thus, they presented late to the hospital. |
|  | Lee HY, Jayalakshmi P, Syed Noori SH.  1993 | Malaysia | Retrospective cross-sectional | 1,2,3 | - Delayed presentation to a health-care facility - Lack of knowledge of appendicitis symptoms and alternative forms of health-care - Delay in diagnosing and managing appendicitis - Lack of infrastructure | Delay in presentation was an important factor. Perforated appendicitis was operated earlier compared to nonperforated cases, due to poor detection of perforation pre-operatively (40%) or as a result of the indifferent attitude towards perforated appendicitis. Women, children, and the elderly with appendicitis were challenging to diagnose. |
|  | Ngim CF, Quek KF, Dhanoa A, *et al.*  2014 | Malaysia | Retrospective cross-sectional | 1,3 | - Lack of knowledge of appendicitis symptoms and alternative forms of health-care - Delay in diagnosing and managing appendicitis | The delay in presenting to our emergency department was probably due to the lack of recognition of appendicitis by family members or primary care providers. |
|  | Tan PH, Teng XX, Gan ZY, *et al.* 2020 | Malaysia | Retrospective cross-sectional | 3 | - Delay in diagnosing and managing appendicitis | Appendicitis in children is commonly misdiagnosed due to their varied presentations. |
